# Essential role of Pin1 *via* STAT3 signalling and mitochondria-dependent pathways in restenosis in type 2 diabetes

**DOI:** 10.1111/jcmm.12082

**Published:** 2013-06-10

**Authors:** Lei Lv, Jiwei Zhang, Lan Zhang, Guanhua Xue, Peng Wang, Qiurong Meng, Wei Liang

**Affiliations:** Department of Vascular Surgery Renji Hospital Shanghai Jiaotong University, College of MedicineShanghai, China

**Keywords:** Pin1, STAT3, mitochondria, restenosis, vascular smooth muscle cells, type 2 diabetes

## Abstract

Type 2 diabetes (T2D) is associated with accelerated restenosis rates after angioplasty. We have previously proved that Pin1 played an important role in vascular smooth muscle cell (VSMC) cycle and apoptosis. But neither the role of Pin1 in restenosis by T2D, nor the molecular mechanism of Pin1 in these processes has been elucidated. A mouse model of T2D was generated by the combination of high-fat diet (HFD) and streptozotocin (STZ) injections. Both Immunohistochemistry and Western blot revealed that Pin1 expression was up-regulated in the arterial wall in T2D mice and in VSMCs in culture conditions mimicking T2D. Next, increased activity of Pin1 was observed in neointimal hyperplasia after arterial injury in T2D mice. Further analysis confirmed that 10% serum of T2D mice and Pin1-forced expression stimulated proliferation, inhibited apoptosis, enhanced cell cycle progression and migration of VSMCs, whereas Pin1 knockdown resulted in the converse effects. We demonstrated that STAT3 signalling and mitochondria-dependent pathways played critical roles in the involvement of Pin1 in cell cycle regulation and apoptosis of VSMCs in T2D. In addition, VEGF expression was stimulated by Pin1, which unveiled part of the mechanism of Pin1 in regulating VSMC migration in T2D. Finally, the administration of juglone *via* pluronic gel onto injured common femoral artery resulted in a significant inhibition of the neointima/media ratio. Our findings demonstrated the vital effect of Pin1 on the VSMC proliferation, cell cycle progression, apoptosis and migration that underlie neointima formation in T2D and implicated Pin1 as a potential therapeutic target to prevent restenosis in T2D.

## Introduction

Peripheral arterial disease (PAD) is a prevalent systemic atherosclerotic disease which can impair a patient's quality of life and even lead to limb loss. Type 2 diabetes is a significant known risk factor for PAD and is increasing in incidence and prevalence [1, 2]. Over the past decade, percutaneous revascularization therapies for the treatment of patients with PAD have evolved tremendously. However, the long-term success of this procedure is limited by recurrent stenosis, especially in patients with T2D. This may be partly because of metabolic derangements that cause endothelial dysfunction [3]. It is also suggested that the poor response to interventions in T2D states appears to be associated with increased inflammatory and proliferative activities that may be driven by increased serum levels of insulin and glucose or by other biochemical aberrancies [4]. Whatever the aetiology of this enhanced restenosis in T2D, induced VSMC proliferation and subsequent migration from the media to the intima contribute significantly to the complex pathophysiological events leading to restenosis [5]. This may, in part, suggest insufficient apoptosis in the diseased tissue [6]. Thus, one current strategy to maintain proper vascular function after angioplasty is to inhibit VSMC proliferation by targeting cell cycle regulation and apoptosis, *e.g.* by drug-eluting stents [7].

A novel post-phosphorylation signalling regulator known as the peptidyl–prolyl cis/trans isomerase Pin1 sits at the crossroads of many signalling pathways controlling cell proliferation and transformation. Pin1 is the only mammalian enzyme known to specifically catalyse the *cis–trans* isomerization of Ser-Pro or Thr-Pro peptide bonds [8, 9]. The effects of the Pin1-induced isomerization on its target proteins are diverse and include altering the stability and localization of the target proteins, as well as modifying their interaction with other proteins [10, 11]. Growing evidence has shown that Pin1 is involved in the pathogenesis of certain cancers and protein folding illnesses like Alzheimer's and Parkinson's disease [12]. We have demonstrated previously that endogenous Pin1 plays an important role in VSMC cell cycle and apoptosis. Specifically, knockdown of Pin1 led to cell cycle arrest in G1 phase and induced apoptosis of VSMCs *in vitro*. We also provided some insights into the molecular mechanisms behind these processes [13]. Intriguingly, we speculated that STAT3 activation might, in part, account for the inhibited growth and enhanced apoptosis of VSMCs transduced with lentiviral siPin1. This finding is consistent with a prior study showing that STAT3 may serve as a possible substrate of Pin1 [14]. However, the role of Pin1 in the formation of intimal hyperplasia in T2D remains largely unknown and whether Pin1 interacts with STAT3 in T2D condition has not been examined. Recently, a study has shed light on the fact that Pin1 induction during neointimal formation may be associated with ROS-mediated VSMC proliferation *via* down-regulation of Nrf2/ARE-dependent HO-1 expression [15]. Although two lines of evidence have linked Pin1 to VSMC proliferation and apoptosis, the function of Pin1 in restenosis in T2D seems to be more complex. In light of these few but important studies, we are interested to investigate the novel role of Pin1 in VSMCs during the development of restenosis in T2D, where anti-Pin1 therapies maybe of value.

In the present study, we sought to determine whether pin1 was responsible for the abnormal VSMC cell cycle and apoptosis as well as injury-induced neointimal growth in T2D and, if so, to explore the underlying mechanism. Our results provided the first evidence that pin1 affected cell cycle and apoptosis through STAT3 signalling and mitochondria-dependent pathways in the VSMCs in T2D condition.

## Materials and methods

### Generation of T2D model

Six-month-old C57BL/6N male mice were purchased from Shanghai SLAC Laboratory Animal Co. Ltd and placed on the HFD (D12492; 60% fat, 20% protein and 20% carbohydrate; 5.24 kcal/g). After 3 weeks of HFD feeding, the mice were injected three times on consecutive days with low-dose STZ (intraperitoneal at 40 mg/kg) to induce partial insulin deficiency. Three weeks after STZ injection, the majority of HFD/STZ-treated mice displayed hyperglycaemia, insulin resistance and glucose intolerance [16]. The normal diet–fed mice were used as non-diabetic controls. All procedures for the use of animals were performed according to the institutional ethical guidelines on animal care, Renji Hospital, Shanghai Jiaotong University, College of Medicine.

### Arterial injury models

Each mouse was anaesthetized by intraperitoneal injection of 50 mg/kg of pentobarbital diluted in 0.9% sodium chloride solution. Guidewire injury of the common femoral artery was performed by three passages of a 0.014-inch guidewire (Radius X-TRa Support PTCA GUIDEWIRE; Radius Medical Technologies, Maynard, MA, USA) [17]. Control sham-operated arteries underwent dissection, temporary clamping without passage of the wire. One hundred microlitres of 20% pluronic gel (Sigma-Aldrich, St. Louis, MO, USA) with or without 300 μg juglone were then applied to the exposed adventitial surface of the common femoral artery right after guidewire injury. Surgery was carried out using a dissecting microscope. Two weeks after guidewire injury, each mouse was cardiac-perfused with 0.9% NaCl solution, followed by perfusion fixation with 4% paraformaldehyde in PBS (pH 7.4) after anaesthesia. The common femoral artery was carefully excised, fixed in 4% paraformaldehyde overnight at 4°C and embedded in paraffin.

### Isolation and culture of VSMCs

Aortas were harvested from C57BL/6N male mice. Adventitia was dissected from media, which was cultured to yield VSMCs, whose phenotype was confirmed by typical morphology and immunohistochemical staining for SMαA. Seven separate isolates of VSMCs were obtained and characterized, derived from a pool of aortas from three individual mice per each genotype. Vascular smooth muscle cells were grown in DMEM containing 10% foetal bovine serum (FBS), and cells between passages 2 and 5 were used for experiments. To mimic a T2D state, post-confluent primary cultured cells were starved for 24 hrs. Then the starvation medium was replaced for 72 hrs with medium containing 10% serum isolated from HFD/STZ-induced T2D mice. Vascular smooth muscle cells grown in medium containing 10% serum isolated from non-diabetic mice for 72 hrs served as control.

### Construction of expression vectors and transfection

The cDNA of the full sequence of Pin1, which was purchased from Guangzhou GeneCopoeia Co., Ltd, were subcloned into the GV166 to obtain GV166-Pin1 (Pin1-overexpressing) construct (GV166:Ubi-MCS-3FLAG-IRES-puromycin, was purchased from Shanghai Genechem Co., Ltd.). GV166-null was used as a control virus. For shRNA sequences, the oligonucleotide was designed from murine pin1 (Genbank accession NM 023371) containing a sense strand of 21 nucleotide sequences followed by a short space (TTCAAGAGA), the reverse complement of the sense strand and six thymidines as a RNA polymerase III transcriptional stop signal. Forward and reverse oligos for Pin1-shRNA (ORF region: 70) were forward: (CTCGAGGGGTGTACTACTTCAATCACATTCAAGAGATGTGATTGAAGTAGTACACCCTTTTTTGAATTC), reverse (GAATTCAAAAAAGGGTGTACTACTTCAATCACATCTCTTGAATGTGATTGAAGTAGTACACCCCTCGAG). The oligonucleotide pairs were designed to contain terminal EcoRI and Xhol restriction sites, and were subcloned into the vector after annealing to generate GV112–Pin1 shRNA vector (GV112:hU6-MCS-CMV-Puromycin, was purchased from Genechem). Lentiviral particles were produced and transfected into cultured VSMCs as described previously [18]. Transfected cells were used for the subsequent experiments 72 hrs after transfection. Both mRNA and protein levels of Pin1 in Pin1-knockdown and Pin1-overexpressing VSMCs were confirmed by real-time PCR, Western blot and immunofluorescence.

### TUNEL assay

TUNEL assay was performed with an apoptosis detection kit (Chemicon, Temecula, CA, USA) according to the manufacturer's protocol. For primary cultured VSMCs, quantifications of positive VSMCs in TUNEL staining were performed by taking images in six random regions of cover slides at a magnification of 20×. The percentages of TUNEL-positive cells per total numbers of cells were counted and analysed. For paraffin-embedded arteries, percentage of apoptosis was calculated by counting the number of apoptotic cells in all cells of each section.

### Immunofluorescent staining

The VSMCs were fixed in 4% paraformaldehyde and permeabilized with 0.1% Triton X-100 at room temperature for 20 min. Thereafter, cells were incubated with anti-α-actin and anti-pin1 antibody (Abcam, Cambridge, UK) and further stained with appropriate TRITC- or FITC-conjugated secondary antibodies (Santa Cruz Biotechnology Inc., Santa Cruz, CA, USA). Nucleus was counterstained with DAPI. Confocal microscopy was performed with the Confocal Laser Scanning Microscope Systems (Leica Microsystems, Wetzlar, Germany).

### RNA isolation and real-time RT-PCR

Total RNA was isolated from VSMCs using Trizol® Reagent (Invitrogen, Carlsbad, CA) according to the manufacturer's instructions. Real-time PCR was performed using a standard TaqMan PCR kit protocol in an Applied Biosystems GraphPad PRISM 4.0 Sequence Detection System using the following PCR primers: Pin1(sense, 5′-GGAGAGGAAGACTTTGAATCTCTGG-3′; antisense, 5′-TGGTTTCTGCATCTGACCTCTG-3′). All data were normalized to β-actin as an internal control.

### Cell migration assay

Vascular smooth muscle cells were plated at an initial density of 1 × 10^5^ cells/ml to form a monolayer. Then, cells were wounded by scraping with a pipette tip to make a gap in the cell monolayer. The images of cell migration were observed at post-scratching hour 0 (immediately after scratching) and 36, and photographed at five marked locations on each dish using a phase-contrast microscope. The number of migrated cells was counted and averaged. All experiments were carried out in triplicate and repeated at least six times.

### Pin1 activity assay

Microdissected arteries (pool of five mice) were placed on ice in a reaction buffer containing 100 mM NaCl, 50 mM HEPES, pH 7, 2 mM DTT and 0.04 mg/ml BSA. The arteries were homogenized using a mini-bead beater (Biospec Products, Inc., Bartlesville, OK, USA) and the supernatant cleared by centrifugation at 12,000 × *g* for 10 min (4°C). Pin1 activity was measured using equal amounts of artery cytoplasmic lysates and α-chymotrypsin using a synthetic tetrapeptide substrate Suc-Ala-Glu-Pro-Phe-pNa (Peptides International, Louisville, KY, USA). Absorption at 390 nM was measured using an Ultrospec 2000 spectrophotometer. The results were expressed as the mean of three measurements from a single experiment and were representative of three independent experiments.

### Cell proliferation assays

Cell proliferation was determined by two methods. Vascular smooth muscle cells (5 × 10^3^ cells/well) were seeded in 96-well culture plates. MTT reagent was added to each well and absorbance of the formazan from each sample was measured at a test wavelength 570 nm at indicated time-points. Vascular smooth muscle cells plated in 12-well plates (4 × 10^4^ cells/well) were then incubated in complete medium containing tritiated [^3^H]-thymidine (1 μCi/ml). Tritiated [^3^H]-thymidine incorporated into trichloroacetic acid-precipitated DNA was measured with a liquid scintillation counter. Each experiment was repeated six times.

### Flow cytometry

Cells were harvested and washed twice with FACS buffer (PBS/1% FCA/0.0025% sodium azide). The pellet was resuspended in 500 ml FACS buffer, and 5 ml of cold ethanol was added. After incubation at 4°C overnight, the ethanol was removed, the pellet was resuspended in FACS buffer and PI solution (500 mg/ml) was added. Then DNA content analysis was performed with a FACScan. Results were expressed as percentage of cells in each cell cycle phase. Cell apoptosis and necrosis were further confirmed by annexin V-PI dual staining flow cytometry. Cells were collected, washed with PBS and suspended in 400 ml binding buffer (10 mM HEPES, 140 mM NaCl, 2.5 mM CaCl2, 0.1% BSA). Annexin V-FITC (5 ml) and PI (5 ml) were then added into each sample. After 30-min. incubation in the dark, cells were analysed by FACScan using Cell Quest Research Software (Becton Dickinson, San Jose, CA, USA).

### Preparation of cytosolic extract

The cytochrome c apoptosis assay kit (Biovision, Mountain View, CA, USA) was used for cytosolic extract in this experiment. Vascular smooth muscle cells were homogenized with the cytosol extraction buffer provided in the kit and then centrifuged at 700 × *g* for 10 min. at 4°C to remove debris. The supernatant was then centrifuged at 10,000 × *g* for 30 min. at 4°C, and stored at −80°C in preparation for Western blot.

### Western blot analysis

Cells were lysed with lysis buffer containing protease inhibitors. Equal amounts of protein aliquots were resolved over SDS–PAGE gels and transferred onto PVDF membrane. Blots were blocked with non-fat dry milk for 1 hr at room temperature and incubated with primary antibodies [Rabbit monoclonal antibodies against Pin1 (1:700), STAT3 (1:400), pTyr705-STAT3 (1:300), STAT1 (1:800), pTyr701-STAT1 (1:500), pSer727-STAT1 (1:500), p16ink4a (1:500), p27kip1 (1:400), caspase-3 (1:200), caspase-9 (1:100), cytochrome c (1:200), Bcl-2 (1:500), Bax (1:500) and VEGF (1:500) were purchased from Abcam. Rabbit polyclonal antibodies against pSer727-STAT3 (1:300), p21waf1/cip1 (1:100) and MMP9 (1:800) were purchased from Abcam. Rabbit polyclonal antibodies against MMP2 (1:500) and Alpha Tubulin (1:1000) were purchased from Santa Cruz Biotechnology Inc.] overnight at 4°C followed by appropriate secondary antibody. The membranes were developed by using ECL reagent protocol. The blots were further probed for Tubulin, which was used as a loading control. The optical density of the immunoradiograms was quantified by densitometric scanning.

### Tissue microarray

Tissue microarray was constructed using the manual tissue arrayer (Beecher Instruments, Silver Spring, MD, USA). Tissue cores were 2 mm in diameter, and the length ranged from 4 to 6 mm. The tissue microarray sections (4 μm) were deparaffinized in xylene and rehydrated using a graded series of ethanol. Streptavidin-biotin-horseradish peroxidase method was used, and the expression of Pin1 was examined with the primary antibodies (Pin1, dilution 1:100) Protein expression of Pin1 was quantified based on the extent of staining (percentage of positive VSMCs).

### Morphometric analysis

Common femoral arteries harvested at 14 days after wire injury were examined histologically for evidence of neointimal hyperplasia using routine haematoxylin and eosin staining. Digital images were collected with light microscopy using an Olympus BHT microscope (Melville, NY, USA) with 40× objective. Six evenly spaced sections through each common femoral artery were morphometrically analysed. Intimal area (I) and medial area (M) were measured (arbitrary units) using ImageJ software (National Institutes of Health, Bethesda, MD, USA).

### Data analysis

Data are presented as mean ± SEM. anova and the paired or unpaired *t-*test was used for statistical analysis as appropriate. *P* < 0.05 was considered statistically significant.

## Results

### Expression levels of Pin1 in vascular tissues

**Fig. 1:**
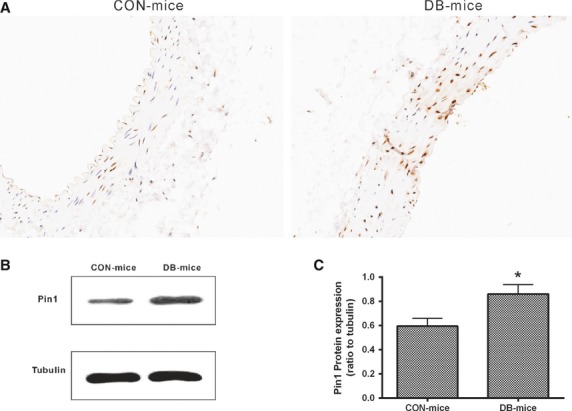
Expression of Pin1 is increased in common femoral arteries of type 2 diabetes (T2D) mice. Immunohistochemistry analysis of Pin1 in common femoral arteries from control (CON-mice) and T2D mice (DB-mice). Representative photographs are shown (magnification, ×200). The brown colour staining represents cellular Pin1 expression (**A**). Artery lysates were analysed by Western blot with antibodies against Pin1 or Tubulin. Tubulin served as a loading control. Representative blots are shown (**B**). Bar graph shows relative densitometric values of Western blots (**C**). Values expressed as mean ± SD from three independent experiments; *P < 0.05 *versus* CON-mice.

**Fig. 2:**
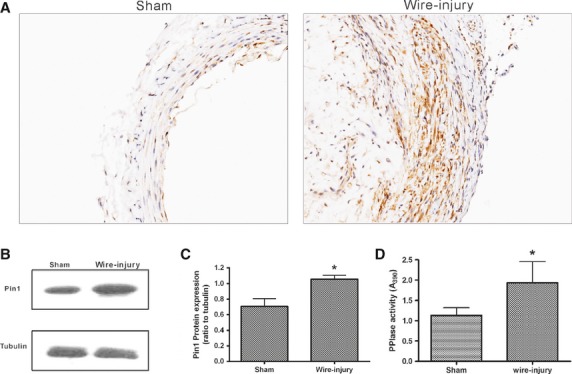
Pin1 is activated after vascular injury in type 2 diabetes (T2D) mice. Immunohistochemistry analysis of Pin1 in common femoral arteries from sham and wire-injured T2D mice on day 14. Representative photographs are shown (magnification, ×200). The brown colour staining represents cellular Pin1 expression (**A**). Artery proteins were extracted from sham and wire-injured T2D mouse common femoral arteries on day 14. Samples were probed with anti-Pin1 monoclonal antibody. Tubulin served as an internal control. Representative blots are shown (**B**). Bar graph shows relative densitometric values of Western blots (**C**). Pin1 activity of artery cytoplasmic lysates from sham and wire-injured T2D mouse common femoral arteries on day 14 is shown (**D**). Pin1 activity was normalized to the total protein concentration of each respective lysate. Values expressed as mean ± SD from three independent experiments; *P < 0.05 *versus* Sham.

We initially determined whether Pin1 was expressed in the vascular wall of HFD/STZ-induced T2D mice. Immunohistochemistry revealed that the expression of Pin1 was clearly observed in the vascular wall of HFD/STZ-induced T2D mice. In contrast, the expression of Pin1 was nearly undetectable in the vascular wall of normal mice (Fig. 1A). Increased expression of pin1 in common femoral arteries of T2D mice was confirmed by Western blot (Fig. 1B and C). This finding let us examine whether Pin1 was associated with intimal hyperplasia in the mouse injury model of T2D. Mild to moderate intimal hyperplasia developed in the mouse common femoral arteries at 7 days in response to wire injury and became more severe at 14 days. So we chose day 14 as our time-point for further evaluation. The expression of Pin1 was markedly increased in the neointima after wire injury in T2D mice (Fig. 2A–C). We then analysed Pin1 enzymatic activity in artery extracts from the sham-operated and wire-injured T2D mice. We found that Pin1 activity was significantly increased in common femoral artery cytoplasmic lysates of the wire-injured T2D mice compared with that of the sham control (Fig. 2D). This suggested that pin1 might be involved in the development of neointimal lesions after wire injury in T2D mice.

### Serum isolated from T2D mice up-regulates Pin1 expression in VSMCs

**Fig. 3:**
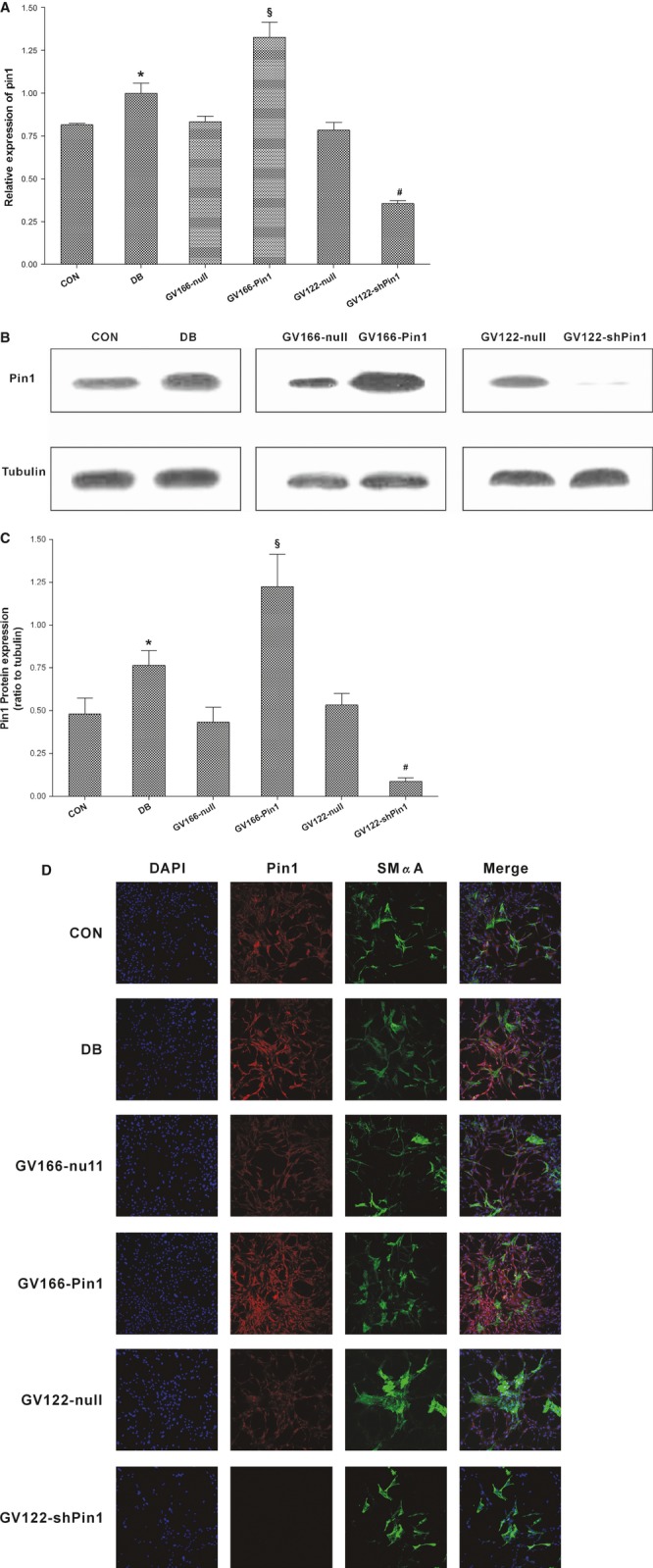
Effects of serum from type 2 diabetes (T2D) mice, GV166-Pin1 and GV122-shPin1 on Pin1 expression in vascular smooth muscle cells (VSMCs). Cells were either cultured in 10% serum from T2D mice (as a control, 10% serum from normal mice was used) or transduced with the indicated lentiviruses. Pin1 mRNA level in VSMCs was determined by quantitative real-time PCR, β-actin was used to compare load amounts (**A**). Cell lysates were immunoblotted with anti-Pin1. Tubulin served as an internal control. Representative blots are shown (**B**). Bar graph shows relative densitometric values of Western blots (**C**). Immunofluorescence double staining of VSMCs with specific antibodies against SMαA (green) or Pin1 (red) was carried out. Nuclei were stained by DAPI (blue), magnification, ×200 (**D**). Values expressed as mean ± SD from three independent experiments; *P < 0.05 *versus* CON; ^§^*P* < 0.05 *versus* GV166-null; ^#^*P* < 0.05 *versus* GV122-null. CON, 10% serum from normal mice; DB, 10% serum from T2D mice.

From the result that Pin1 was up-regulated in the vascular wall of HFD/STZ-induced T2D mice, the question arose as to how the expression of Pin1 was regulated in a condition that happened in T2D *in vitro*. Culture in 10% serum isolated from HFD/STZ-induced T2D mice was intended to mimic T2D condition. As shown in Figure 3, Pin1 was increased in VSMCs cultured in 10% serum isolated from HFD/STZ-induced T2D mice. Pin1 protein was found in both cytoplasm and nucleus, with the latter being more condensed.

### Effective overexpression or knockdown of Pin1 in VSMCs

To better understand the roles of Pin1 in stenosis in T2D, we transfected VSMCs with either GV166-Pin1 or GV166-null to investigate its effect. Results of RT-PCR revealed that GV166-Pin1–transfected cells suffered a dramatic increase in Pin1 mRNA, while GV166-null did not (Fig. 3A). Results of Western blot demonstrated that Pin1 protein expression was remarkably higher in VSMCs transfected with GV166-Pin1 than the control (Fig. 3B and C). As the fluorescence microscope showed (Fig. 3D), the fluorescent staining was stronger in intensity and larger in number in cells transfected with GV166-Pin1. All the data above demonstrated that the GV166-Pin1 was effective in overexpressing Pin1 in VSMCs. Likewise, the efficiency of lentiviral shRNA for Pin1 was confirmed by real-time RT-PCR, Western blot and fluorescent staining. Endogenous Pin1 expression of VSMCs was overwhelmingly suppressed (Fig. 3), indicating that Pin1 was knocked down effectively.

### Role of Pin1 in VSMC growth, apoptosis and migration

**Fig. 4:**
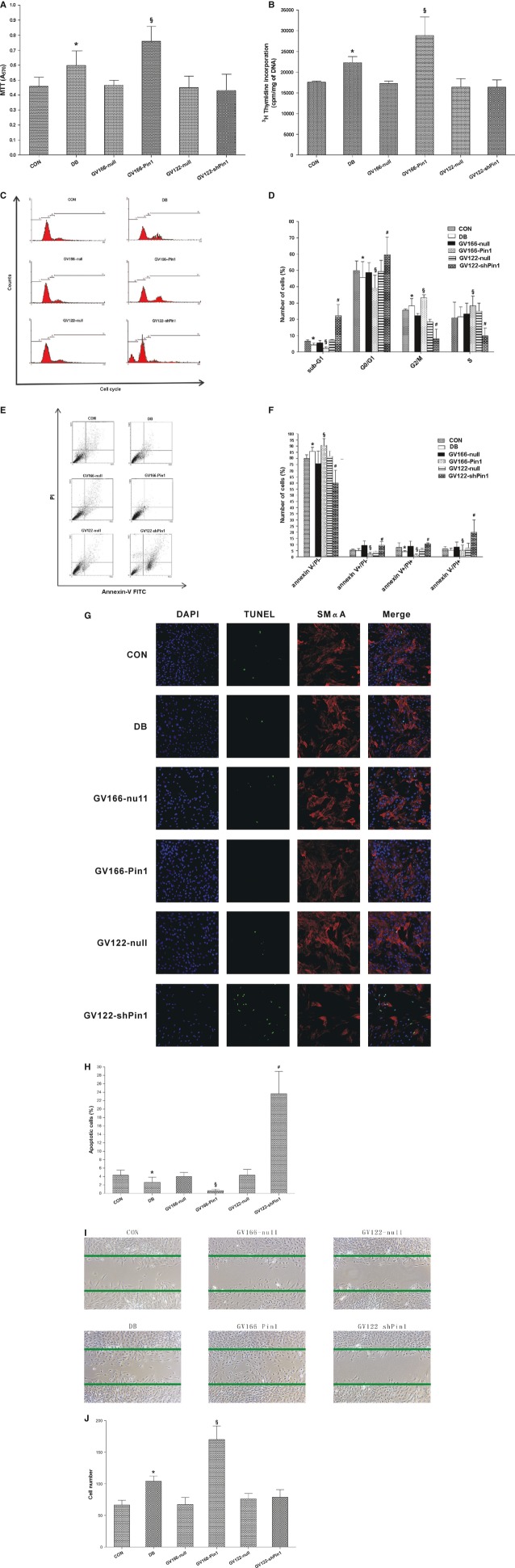
Effects of Pin1 on proliferation, cell cycle progression, apoptosis and migration in vascular smooth muscle cells (VSMCs). VSMCs were cultured and infected as described in Figure 3. Cell proliferation was assessed by both the MTT assay (**A**) and [^3^H]-thymidine incorporation analyses (**B**). Cell cycle analysis was quantified by PI staining followed by flow cytometry analyses. M1, M2, M3 and M4 indicate sub G1, G0/G1, S and G2/M phases respectively (**C**). Bar graphs represent the mean ± SEM of three independent experiments (**D**). Cell apoptosis was detected by annexin V-FITC/PI staining (**E**) followed by flow cytometry analysis and TUNEL staining (**G**). The proportion of live cells (annexin V−/PI−), early apoptotic cells (annexin V+/PI−), late apoptotic/necrotic cells (annexin V+/PI+) and dead cells (annexin V−/PI+) was calculated for comparison (**F**). The TUNEL-positive cells were stained green, VSMCs were stained for SMαA and shown in red. Nuclei were stained blue by DAPI. The percentages of apoptotic nuclei were calculated by determining the number of DAPI-stained nuclei that were also positive for TUNEL staining. Approximately, 100 nuclei cells were counted in randomly chosen fields per region (**H**). VSMC migration was determined by a standard wound healing assay (**I**). Bar graphs represent the mean ± SEM of three independent experiments (**J**). *P < 0.05 *versus* CON; ^§^*P* < 0.05 *versus* GV166-null; ^#^*P* < 0.05 *versus* GV122-null. CON, 10% serum from normal mice; DB, 10% serum from type 2 diabetes mice.

As demonstrated in Figure 4, 10% serum from HFD/STZ-induced T2D mice promoted proliferation and inhibited apoptosis of VSMCs. Forced expression of Pin1 in cells resulted in faster cell proliferation rates when compared with cells transfected with the control. In contrast, Pin1 deletion by lentivirus-mediated transfer negatively impacted the proliferation of VSMCs (Fig. 4A). Using [^3^H]thymidine incorporation in VSMCs, a similar proliferation status was observed (Fig. 4B). Pin1-forced expression displayed less cells in G0/G1 phase and relatively high percentages of populations in S and G2/M phases. Conversely, lenti-shPin1 impaired VSMC cycle, thus increasing the percentage of cells in G0/G1 and reducing the percentage of cells in S and G2/M phases (Fig. 4C and D). It was noteworthy that early apoptotic cells were elevated significantly following lenti-shPin1 transfection (Fig. 4E and F). In accord, apoptosis was blocked or induced by overexpressing or silencing Pin1 as demonstrated by TUNEL assay (Fig. 4G and H). In *in vitro* scratch assay, 10% serum from HFD/STZ-induced T2D mice enhanced VSMC migration. In addition, Pin1-overexpressing cells revealed significant migration advantages compared with control cells, whereas knockdown of Pin1 showed reduced migratory capacity (Fig. 4I and J).

### STAT3 activation is mediated by Pin1

**Fig. 5:**
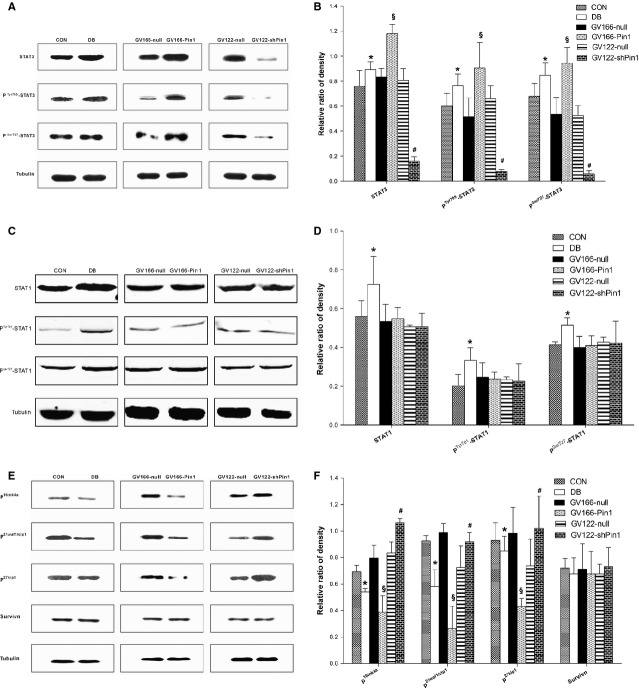
Pin1 modulates cell growth and cell cycle progression by interfering with the STAT3 signalling pathway. Vascular smooth muscle cells (VSMCs) were cultured and infected as described in Figure 3. The levels of STAT3, pTyr705-STAT3, pSer727-STAT3, STAT1, pTyr701-STAT1 and pSer727-STAT1 were quantified by Western blot analysis (**A** and **C**). The levels of some STAT3 downstream targets (p16ink4a, p21waf1/cip1, p27kip1 and survivin) in VSMCs were further observed by Western blot (**E**). Tubulin served as an internal control. Bar graph shows relative densitometric values of Western blots (**B**, **D** and **F**). Data represent mean ± SEM of three independent experiments. *P < 0.05 *versus* CON; ^§^*P* < 0.05 *versus* GV166-null; ^#^*P* < 0.05 *versus* GV122-null. CON, 10% serum from normal mice; DB, 10% serum from type 2 diabetes mice.

To elucidate whether STAT3 activation was regulated by Pin1 in T2D condition, we examined the protein levels of STAT3 as well as phosphorylations of STAT3 at Tyr705 and Ser727 in VSMCs by means of Western blot analysis. As shown in Figure 5A and B, the level of STAT3, JAK-phosphorylated form of STAT3, indicated as pTyr705-STAT3 and PKC-phosphorylated form of STAT3, indicated as pSer727-STAT3, were enhanced in VSMCs cultured in 10% serum from HFD/STZ-induced T2D mice. Overexpression of Pin1 markedly increased STAT3, pTyr705-STAT3 and pSer727-STAT3 level, whereas the knockdown of Pin1 resulted in a significant decrease in STAT3, pTyr705-STAT3 and pSer727-STAT3 level. As shown in Figure 5C and D, the level of STAT1 and phosphorylation of STAT1 on both Ser727 and Tyr701 were elevated in VSMCs cultured in 10% serum from T2D mice. Nevertheless, no significant change in the level of STAT1, pTyr701-STAT1 or pSer727-STAT1 was observed in Pin1-overexpressed or -silenced VSMCs.

### Pin1 is associated with the modulation of some downstream STAT3 targets

We further examined the expression of various apoptosis and cell cycle regulatory proteins known to be downstream targets of the STAT3 pathway. Ten percent serum from HFD/STZ-induced T2D mice induced down-regulation of p16ink4a, p21waf1/cip1 and p27kip1 in VSMCs. However, no change was seen at the protein level for survivin. Consistent with this finding, we found that the expression of p16ink4a, p21waf1/cip1 and p27kip1 was significantly attenuated by Pin1 overexpression, but increased by Pin1 knockdown. Nevertheless, the knockdown or overexpression of Pin1 did not cause a remarkable change in survivin level (Fig. 5E and F).

### Pin1 suppresses VSMC apoptosis in association with cytochrome c release and caspase activation

**Fig. 6:**
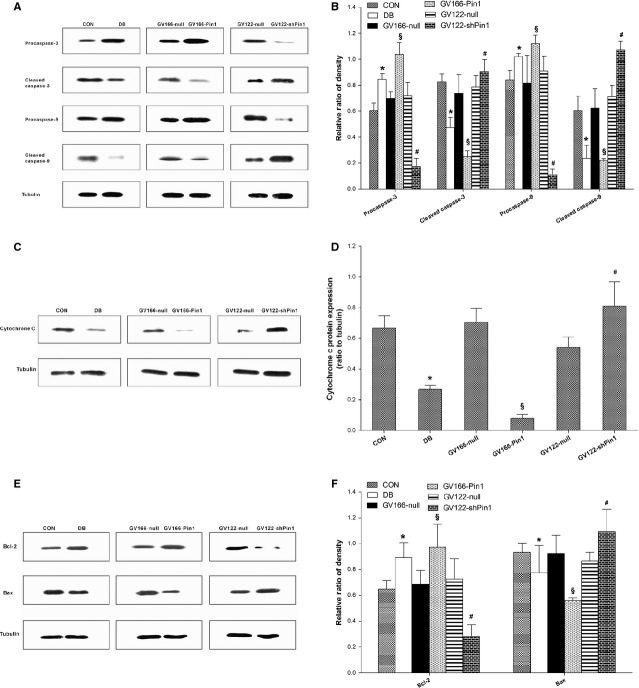
The caspase- and mitochondria-dependent pathways are involved in Pin1-mediated cell growth and apoptosis. Vascular smooth muscle cells (VSMCs) were cultured and infected as described in Figure 3. The levels of procaspase-3, procaspases-9 and the large subunits of the cleaved forms of caspase-3 and caspases-9 were quantified by Western blot analysis (**A**). While, in parallel, the levels of cytosol cytochrome c in VSMCs were performed by Western blot analysis (**C**). The expression of Bax and Bcl-2 was also examined by Western blot analysis (**E**). Tubulin served as an internal control. Bar graph shows relative densitometric values of Western blots (**B**, **D** and **F**). Data represent mean ± SEM of three independent experiments. *P < 0.05 *versus* CON; ^§^*P* < 0.05 *versus* GV166-null; ^#^*P* < 0.05 *versus* GV122-null. CON, 10% serum from normal mice; DB, 10% serum from type 2 diabetes mice.

To explore whether Pin1 suppressed VSMC apoptosis through caspase-dependent pathway in T2D condition, we examined the activation of caspase-3 and -9 by Western blot analysis. It was well known that mitochondria-mediated activation of caspase-3 and -9 involved the releases of cytochrome c. Next, the level of cytochrome c in the cytosol was tested by Western blot. As expected, our results indicated that the activation of caspase-3 and -9 was inhibited in VSMCs cultured in 10% serum from HFD/STZ-induced T2D mice. The treatment with 10% serum from HFD/STZ-induced T2D mice decreased cytoplasmic level of cytochrome c. Furthermore, the levels of active form of caspase-3 and -9 were markedly inhibited in Pin1-overexpressing VSMCs while strongly stimulated in VSMCs silenced for Pin1. Overexpression of Pin1 blocked the release of cytochrome c while knockdown of Pin1 triggered up-regulation of cytosol cytochrome c (Fig. 6A–D).

### Pin1 represses VSMC apoptosis coincided with increase in Bax/Bcl-2 ratio

To delineate the signalling events involved in repressed VSMC apoptosis by Pin1 in T2D condition, we examined the potential role of Pin1 in regulating the expression of Bax and Bcl-2. Remarkably, antiapoptotic protein Bcl-2 expression was increased. In contrast, the protein abundance of Bax, a pro-apoptotic member, was decreased in VSMCs cultured in 10% serum from HFD/STZ-induced T2D mice. Moreover, Bax/Bcl-2 ratio in VSMCs overexpressed for Pin1 was dramatically decreased but elevated in VSMCs silenced for Pin1 (Fig. 6E and F). This further substantiated our conclusion that Pin1 inhibited VSMC apoptosis *via* the mitochondrial apoptotic signalling pathway.

### Induction of VEGF but not MMP2 and MMP9 by Pin1

**Fig. 7:**
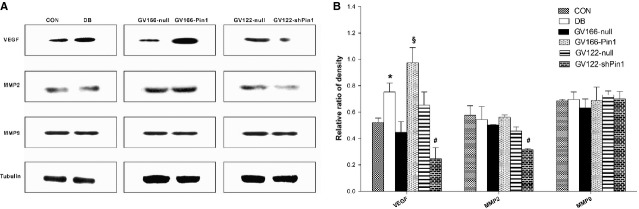
Pin1 regulates cell migration by modulating VEGF expression. Vascular smooth muscle cells (VSMCs) were cultured and infected as described in Figure 3. The levels of VEGF, MMP2 and MMP9 were determined by Western blot analysis (**A**). Tubulin served as an internal control. Bar graph shows relative densitometric values of Western blots (**B**). Data represent mean ± SEM of three independent experiments. *P < 0.05 *versus* CON; ^§^*P* < 0.05 *versus* GV166-null; ^#^*P* < 0.05 *versus* GV122-null. CON, 10% serum from normal mice; DB, 10% serum from type 2 diabetes mice.

To better understand the mechanisms of Pin1 on VSMC migration in T2D condition and to reveal downstream events of STAT3 signalling that were involved in the regulation of cell migration, we examined the expression of various migration regulatory proteins by Western blot analysis (Fig. 7). Our results pointed out that the expression of VEGF was elevated in VSMCs cultured in 10% serum from HFD/STZ-induced T2D mice. Enforced expression of Pin1 led to increased VEGF while depletion of Pin1 enhanced the degradation of VEGF in VSMCs. However, no significant change in MMP9 secretion was seen in VSMCs treated with either GV166-Pin1 or GV112–Pin1 shRNA. Of note, diminishing Pin1 expression affected MMP2 level, however, 10% serum from T2D mice and Pin1 overexpression were assumed to have no association with MMP2 level.

### Pin1 deficiency decreases injury-induced arterial neointima formation

**Fig. 8:**
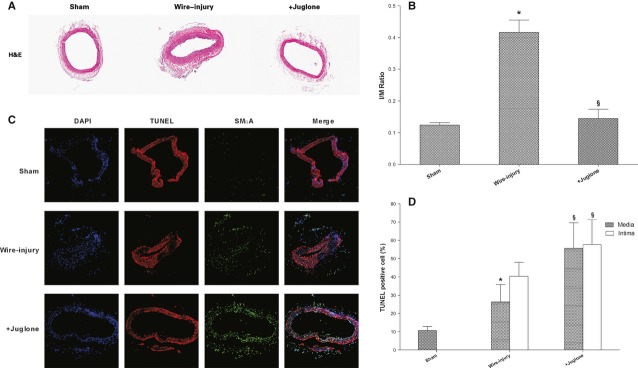
Topical use of juglone inhibits neointimal hyperplasia following arterial injury in a mouse model of type 2 diabetes (T2D). Wire-mediated vascular injury was produced in T2D mice. The common femoral arteries were excised at 14 days after injury. Sample sections were stained with haematoxylin and eosin and neointimal formation was evaluated. Representative photographs of haematoxylin and eosin staining were shown, magnification, ×40 (**A**). Bar graphs show the I/M ratio (**B**) quantified by Image-Pro Plus 6.0 software. Immunohistochemical analysis of arterial sections for apoptosis by TUNEL assay was shown (**C**). The TUNEL-positive cells were stained green, smooth muscle alpha-actin was stained red and nuclei were stained blue by DAPI. Quantitation of the number of apoptotic vascular smooth muscle cells (VSMCs) in the medial or intima layer of common femoral artery was shown (**D**). DAPI-stained nuclei that were also positive for TUNEL staining were counted from randomly selected images and expressed as percentage of TUNEL-positive cells as compared with total cells. Significantly more apoptotic VSMCs were found in the juglone-treated samples than in the plain gel treatment control. Data are expressed as mean ± SEM. Wire-injury: injury alone, + juglone: wire injury plus application of topical use of juglone.*P < 0.05 *versus* sham; ^§^*P* < 0.05 *versus* wire-injury.

On the basis of our observation that pin1 was up-regulated after vascular injury in a HFD/STZ-induced mouse model of T2D and recognition of the inducible effect of pin1 deficiency on VSMC apoptosis *in vitro*, we suggested that juglone, a Pin1 inhibitor, suppressed *in vivo* neointimal formation induced by wire injury in T2D. With application of juglone *via* pluronic gel onto injured common femoral artery, the neointima formation was significantly reduced as compared to the plain gel–treated control (Fig. 8A). With computerized image analysis, the areas of neointima and media layers for each section were calculated. The present study demonstrated a 67% reduction on the area ratio of I/M by juglone as compared with control (Fig. 8B). Next, TUNEL assay was performed to determine the level of cellular apoptosis in neointima or media. Arterial walls in the sham control contained few nucleus-stained cells. More apoptotic cells appeared in the juglone treatment group compared with plain gel treatment group (Fig. 8C). As shown in Figure 8D, the apoptosis rates in both intima and media in juglone treatment group were significantly increased when compared with the plain gel treatment group (*P* < 0.05).

## Discussion

Despite the huge amount of data accumulated so far, much attention has been devoted to the roles of Pin1 played in ageing, cancer and Alzheimer disease [12]. Neither the identity of Pin1 in hyperproliferative vascular disorders, such as arteriosclerosis and restenosis, nor the molecular mechanism of Pin1 function in these diseases was clear. Very recently, Pin1 induction was described in neointimal formation, which was significantly suppressed after intraperitoneal injection of juglone [15]. This phenomenon was in agreement with our previous report that down-regulation of Pin1 in VSMCs induced cell cycle arrest and apoptosis *in vitro*. These pieces of evidence suggested that Pin1 might be critical in the pathological process of restenosis. Strikingly, our present results found that 10% serum from T2D mice stimulated proliferation, inhibited apoptosis, enhanced cell cycle progression and promoted migration of VSMCs, coinciding with Pin1 up-regulation. With the aim of clarifying the mechanism of Pin1 during neointimal hyperplasia in T2D condition, lentiviral strategies for overexpression or knockdown of Pin1 were used. We then asked whether Pin1 played a key role in restenosis in T2D mice. Surprisingly, wire injury induced neointimal hyperplasia in the mouse model of T2D with activation of Pin1. We succeeded in attenuating neointimal hyperplasia in the mouse model of T2D by gently spreading 20% pluronic gel containing juglone around the outside of the common femoral arteries, which led to apoptosis of VSMCs in the arterial walls.

A number of clinical findings suggest that Pin1 has a critical role in the genesis of many human malignancies. It has been also reported that Pin1 may act as an initial signal that subsequently exaggerates proliferation of several human cell lines [12, 19]. Similar to human, it has been established that Pin1 induces proliferation and tumorigenicity of some mouse cells both *in vitro* and *in vivo* [20, 21]. In the present study, we generated a non-genetic mouse model of T2D by employing the combination of HFD and three 40 mg/kg STZ injections, given that this mouse model more closely mimicked the metabolic profile that characterized T2D in human beings [16]. Analysis of the common femoral arteries of T2D mice revealed high levels of Pin1 relative to healthy controls. Insulin had been reported to induce proliferation of VSMCs from human beings and non-human beings [22]. It was known that insulin-resistant patients, such as those affected by obese T2D at the beginning of diabetes natural history, showed increased levels of circulating insulin [23]. In addition, undoubtedly high glucose level could induce VSMC proliferation. Conceivably, these findings might explain why VSMCs cultured in 10% serum from T2D mice reflected an imbalance between growth and apoptosis.

It was reported that the regulation of STAT3 activity might be involved in the pathogenic mechanisms of T2D. Hepatic STAT3 signalling had been identified to be essential for normal glucose homoeostasis and disrupting this signalling pathway could contribute to the onset and progression of diabetes [24, 25]. Jeong *et al*. [26] recently identified a Ser^727^ phosphorylation-dependent and Tyr^705^ phosphorylation-independent STAT3 activation mechanism in the modulation of insulin signalling. Derek *et al*. [27] reported that knockdown of hepatic SirT1 increased STAT3 acetylation and STAT3 phosphorylation (Y705), thus decreased endogenous and insulin-stimulated glucose production and reduced fasting hyperglycaemia in a rat model of T2DM. Jie *et al*. [28] demonstrated that high concentration of insulin treatment resulted in a reduction of total and phosphorylated STAT3 protein. Interestingly, we showed that in T2D condition, STAT3 was a downstream effector of Pin1 and STAT3 expression could be positively mediated by Pin1, either directly or indirectly. In general, Tyr^705^ phosphorylation, typically by the Janus kinase kinases, is involved in STAT3 dimerization and activation, whereas Ser^727^ phosphorylation is believed to modulate STAT3 activity. A previous report indicated that Pin1 up-regulated STAT3 transcriptional activity *via* Ser727 residue of STAT3 [15]. Nevertheless, our present results showed that in addition to the phosphorylation of STAT3 at Ser727, phosphorylation of STAT3 at Tyr705 was also stimulated by Pin1 up-regulation. The discrepancy between the previous report and ours may be because of cell-type specificity of Pin1 action.

STAT1, which shares the highest homology with STAT3 among the STAT family members [29], has been implicated in cell growth deregulation and disturbed immune function, *i.e*. disorders that are pertinent to malignancy [30]. Therefore, in addition to STAT3, the level of STAT1 and phosphorylated STAT1 (the phosphorylation of STAT1 at residue Tyr701 and Ser727) was also subjected to Western blot analysis to determine specificity. It has been suggested that high glucose can cause the activation of STAT1 [31, 32]. Consistently, we found that the exposure of VSMCs to 10% serum from T2D mice caused increased STAT1 and phosphorylation of STAT1. However, forced or silenced Pin1 did not alter the expression of STAT1 or phosphorylated STAT1, thus suggesting that STAT3, but not STAT1, might be mediated by Pin1.

As described by numerous studies, STAT3 activation was implicated in modulating the activity of downstream mediators, hence playing a key role in cell survival, proliferation, and differentiation. Siggins *et al*. [33] reported that alcohol enhanced G-CSF-associated STAT3- p27kip1 signalling, which impaired granulopoietic progenitor cell proliferation by inducing cell cycling arrest and facilitating their terminal differentiation during the granulopoietic response to pulmonary infection. Zhang *et al*. [34] confirmed that both p16ink4a and p21waf1/cip1 were significantly induced by AR-42. This together with a decrease in activation of STAT3 resulted in G_1_ and G_2_ cell cycle arrest. A recent report indicated that STAT3 activation was critical for VZV, a non-oncogenic herpesvirus, *via* a survivin-dependent mechanism [35]. In this regard, we evaluated these essential downstream targets of STAT3. Our present data showed that STAT3 regulated by Pin1 modulated the p27kip1, p16ink4a and p21waf1/cip1 expression, but had no obvious effect on expression of survivin.

To further explore the molecular mechanism by which Pin1 affected cell apoptosis, we examined a possible link to a mitochondria-mediated, caspase-dependent apoptotic pathway. Intrinsic apoptosis pathway is regulated by Bcl-2 family members. Bcl-2 is one of the pro-survival proteins, while Bax is a pro-apoptotic protein [36]. Dysregulation of these proteins causes the release of cytochrome c from the mitochondria to the cytosol, thus activating caspase-9, prompting the activity of caspase-3 and resulting in apoptosis of cells [37]. As reported, treatment of HK-2 cells with high glucose and angiotensin II increased the protein–protein association between p-p66Shc and Pin1 in the cytosol, and with cytochrome c in the mitochondria [38]. A recent report demonstrated that blockade of Pin1 led to cleavage and mitochondrial translocation of Bax and caspase activation [39]. In aggregate, our present data suggested that Pin1 regulated cell proliferation by enhancing the resistance to apoptosis through dysfunction of the Bax/Bcl-2/cytochrome c/caspases-9 and -3 signalling pathway.

Our results also showed that 10% serum from T2D mice promoted VSMC migration, while up-regulating Pin1 expression. This effect was more pronounced in Pin1 overexpression, whereas its knockdown had the opposite effect, confirming a Pin1-specific effect. Cohen *et al*. [40] observed that inhibiting Pin1 activity increased p53 activity towards its target genes MMP-9 and MMP-2, thus confirming the role of Pin-1 in the regulation of trophoblast invasiveness. Other studies have indicated that Pin1 increased the transcriptional activity and protein level of VEGF [41, 42]. To understand the possible mechanism responsible for increased migration in T2D, we looked for changes in the expression levels of VEGF, MMP2 and MMP9. In the present study, no alteration in expression of MMP9 could be observed either by increasing or decreasing Pin1 expression. Only by diminishing Pin1 expression could MMP2 level be affected. Ten percent serum from T2D mice and Pin1 overexpression were assumed to have no association with MMP2 level. Our data supported that Pin1 was an essential positive regulator in the VSMC migration process *via* mediating VEGF expression. Nevertheless, we could not exclude the possibility that Pin1 also modulated other mechanisms, including MMP2 and MMP9 regulation that promoted VSMC migration in the condition of T2D. Thus, the precise mechanism remains to be further investigated.

Finally, we elucidated the role of Pin1 that participated in neointimal formation after vascular injury in T2D mice. Our data showed that the I/M ratio was significantly reduced in juglone-treated common femoral arteries compared with plain gel–treated controls. Moreover, topical use of juglone significantly induced the apoptotic VSMCs in the artery walls after wire injury. These results substantiated our *in vitro* findings and strongly supported the notion that down-regulation of Pin1 conferred protection against injury-induced pathological vascular restenosis.

Taken together, we found that 10% serum from T2D mice and Pin1 overexpression markedly promoted growth, repressed apoptosis, stimulated cell cycle progression and migration of VSMCs, whereas the opposite effects were observed in VSMCs depleted of Pin1. Mechanistically, STAT3 signalling and mitochondria-dependent/caspase-dependent pathways played critical roles in Pin1-mediated cell cycle regulation and apoptosis of VSMCs in T2D condition. In addition, VEGF significantly contributed to VSMC migration mediated by Pin1 in T2D condition. To determine whether our *in vitro* findings had any physiological relevance, we evaluated the *in vivo* effect of Juglone on neointima formation in the guidewire-injured common femoral arteries in T2D mice. Our results confirmed the beneficial effects of Pin1 degradation. This study suggested that Pin1 inhibitors or depletion of Pin1 protein serve as potential therapeutic candidates that warrant further investigation regarding their potential use in the prevention of restenosis in T2D.
